# Normal Weight 6–12 Years Boys Demonstrate Better Cognitive Function and Aerobic Fitness Compared to Overweight Peers

**DOI:** 10.3390/medicina58030423

**Published:** 2022-03-14

**Authors:** Vaida Borkertienė, Laura Valonytė-Burneikienė

**Affiliations:** 1 Department of Health and Rehabilitation, Lithuanian Sports University, LT-44221 Kaunas, Lithuania; 2 Biomedicine Science Faculty, Panevežys University of Applied Sciences, LT-35200 Panevėžys, Lithuania; lauravalonyte@yahoo.com

**Keywords:** children, oxygen uptake, weight, cognitive function

## Abstract

*Background and Objectives:* This study evaluated and compared the cognitive function (CF) and aerobic fitness (AF) of 15 normal-weight (NW) and 15 overweight (OW) children, aged 6–12 years. In addition, the relationship between CF and AF was evaluated. *Materials and Methods*: The ANAM4 battery was used to evaluate CF, and a constant treadmill walking exercise (6 km/h for 6 min) and a progressive treadmill exercise (modified Balke test) were used to assess pulmonary oxygen uptake (VO_2_). *Results*: The OW children displayed worse attention and visual tracking (88.95 ± 4.45% and 93.75 ± 3.16%), response inhibition (90.27 ± 1.54% and 93.67 ± 2%), and speed of processing (93.65 ± 1.5% and 94.4 ± 1.54%) than the NW children (*p* < 0.05). The VO_2_ max was higher and the time constant of VO_2_ kinetics was shorter in NW children (56.23 ± 3.53 mL/kg/min and 21.73 ± 1.57 s, respectively) than in OW children (45.84 ± 1.89 mL/kg/min and 33.46 ± 2.9 s, respectively; *p* < 0.05). *Conclusion*: The OW children aged 6–12 years demonstrated poorer CF and lower AF than their NW peers. An association between AF and CF indicators was identified in both groups.

## 1. Introduction

It has been almost two years with the world living in pandemic restrictions. Working from home, studying has become the new normal. The World Health Organization released new recommendations for physical activity and sedentary behavior in 2020 [1]. At least 60 min per day of moderate to vigorous, mostly aerobic, physical activity is recommended for children to keep healthy musculoskeletal and cardiovascular systems. It is recommended to spend as little as possible sedentary time, because sedentary behavior is associated with the following poor health outcomes: poorer sleep quality, increased adiposity, decreased cardiovascular and musculoskeletal fitness, and also poorer mental health. Runacres et al. [2] found that children increased their sedentary time by more than five hours per day during the pandemic period. Stavridou et al. [3] reported that during the pandemic period overweight and obesity among children increased. A total of 1.27 million new childhood obesity cases were captured in the US during 2020 year [4]. As the number of overweight and obesity is increasing, it is important to understand the negative impact of obesity not only for physical, but also for mental health.

Peak oxygen uptake (VO_2_ peak) has become the most researched variable in pediatric exercise science, and the kinetics of oxygen uptake (VO_2_) is an important parameter of aerobic fitness (AF) in children [5]. VO_2_ peak relative to body weight was lower in obese than normal-weight (NW) children aged 9–14 years [6], and lower estimated VO_2_ max was detected in overweight (OW) children aged 8–16 years compared with their non-OW peers [7]. Obese children aged 10–13 years had higher absolute VO_2_ peak, lower VO_2_ peak corrected for mass, and lower VO_2_ peak corrected for fat-free mass, compared with their NW peers [8]. Furthermore, a higher absolute VO_2_ peak, but lower relative VO_2_ peak, was detected in obese compared with non-obese children aged 10–12 years, while performing both incremental and supramaximal constant-load verification tests [9].

Some studies have reported that OW children demonstrate similar VO_2_ on-kinetics during submaximal exercise [10,11,12]. However, more recent studies reported that OW children and obese adolescents displayed slower oxygen uptake kinetics than NW children while performing both medium and vigorous exercise [13,14,15].

Significant changes in brain structure and function occur during infancy and childhood. OW and obesity have a negative impact on cognitive function (CF), and CF is poorer between the OW and obese populations than the non-obese population [16]. The term CF covers multiple mental abilities, including specific academic skills, scores and school achievements, thinking, problem solving, memory, language, intelligence quotient, and others [17].

OW or obese children demonstrate lower self-esteem, depression, and other emotional consequences [18], which may have an impact on their cognitive achievements in school. Children with a high body mass index have meaningfully lower scores in mathematics and reading in the third grade [19,20]. However, some researchers reported that there are no associations between body mass index and academic results [21,22], while others reported a negative association between higher body mass and academic results for boys, but not girls [23].

The rate of executive dysfunction in obese and OW children was twice that in the normal weight population [24]. Obese or OW youth demonstrated slower cognitive processing speed on cognitive functioning compared with NW peers. In addition, NW children aged 8–11 years displayed better CF than peers with lower or higher weights [25]. One of the most important cognitive skills that develop during childhood is working memory, and children with a higher body mass index are characterized as having worse working memory than NW children [26].

Our previous research revealed that OW youth demonstrate worse CF and AF than NW and sport-trained youth [27]. In a further investigation of CF and AF, here, we analyzed data from children aged 6–12 years who participated in our study. The aim of this study was to evaluate and compare cognitive function and aerobic fitness of normal weight and overweight children, aged 6–12 years.

## 2. Experimental Section

Participants fifteen healthy (15) NW and 15 OW boys aged 6–12 years participated in this study (Table 2). OW status was established using age, height, and weight (BMI between 84th and 95th percentiles—overweight for 6–15 years old). The study was conducted in accordance with the Declaration of Helsinki, and the protocol was approved by the Kaunas Regional Ethics Committee, Nr BE-2-27, 24 May 2017. Written informed consent was obtained from the parents of the participants and verbal agreement was obtained from the participants. Participants of NW and OW groups did not have any specific physical education, except for physical education lessons in school (2 times/week, 45 min/time), which are obligatory for all healthy pupils in Lithuania. Potential participants were excluded from the study if they had musculoskeletal problems, chronic diseases, epilepsy, asthma, heart disease, or diabetes. The characteristics of the participants are presented in Table 2.

### 2.1. Measurements

We used a TBF-300 body composition analyzer to measure body mass to the nearest 0.1 kg beam, and a Stadiometer was used to measure height to the nearest 0.01 m. Body mass index (BMI) was calculated as body mass (kg) divided by height squared (m^2^).

An Oxycon Mobile portable telemetric system (Munich, Germany) was used to record the pulmonary gas exchange parameters during exercise. The gas analyzer and the flow-volume sensor were calibrated before each test session using the automatic calibration procedure provided by Jaeger.

A Polar system was used to measure heart rate (HR). Children wore a pediatric wireless chest strap containing a sensor. The heart’s electrical signals were used to detect and record HR data.

Progressive treadmill exercise (PTE). We used a modified Balke test [28] to collect oxygen uptake data. Each participant stood on the treadmill for 1 min to register their initial gas exchange, then the participant began a stepping protocol (3-min; 3 km/h; 0% grade). Thereafter, the speed of stepping increased to 6 km/h and the grade of the treadmill increased from 0% to 2%. From 4 min onward, the grade of the treadmill increased by 2% every minute of the test, until the participant reached exhaustion. Participants were verbally encouraged to make their maximum effort throughout the test. The test was performed 2 times with 45 min brake between fulfillments. The average of the results were used for the processing of statistical data. Oxygen uptake data was recorded during the test and while the participant was resting.

**Figure 1:**
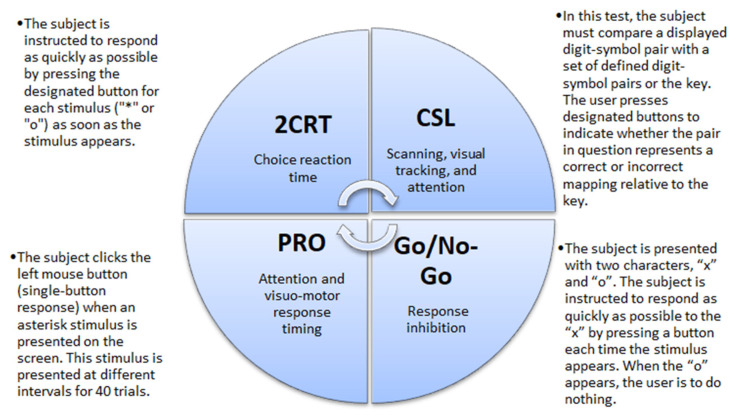
Descriptions of the cognitive tests. 2CRT—2-Choice Reaction Time test; CSL—Code Substitution–Learning, PRO—Simple Reaction Time.

CF was evaluated using the Automated Neuropsychological Assessment Metrics Version 4 (ANAM4). Data were recorded for four tests: 2-Choice Reaction Time test (2CRT), Code Substitution–Learning (CSL), Go/No-Go test, and Simple Reaction Time (PRO) (Figure 1). From all recorded data we analyzed the percent of correct responses. Participants learned to do tests twice, and then the real tests were recorded for evaluation. Participants took about 20–15 min to complete the tests [29].

### 2.2. Study Design

Participants were asked to come to the laboratory twice.

The first time, participants performed the PTE–Balke test. The treadmill angle rose 2% every min, and the speed was constant during the test—6 km/h. Participants were asked and encouraged to continue the exercise until exhaustion. The test was performed 2 times with 45 min brake between fulfillments. The average of the results were used for the processing of statistical data. Oxygen uptake data was recorded during the test and while the participants were resting. All oxygen uptake parameters were recorded during the first visit.

The CF of participants was tested during the second visit. The participants initially performed two trials to learn how to do cognitive tests, and thereafter the four tests were conducted. Participants took about 20–30 min to complete the CF battery.

### 2.3. Data Analysis

To determinate VO_2_ parameters and cognitive function results, we used formulas given in Table 1.

### 2.4. Statistical Analysis

We used SPSS version 22.0 (IBM Corp., Armonk, NY, USA) for statistical analysis. The Kolmogorov–Smirnov test was used to assess the normal distribution of the data; we found all data normally distributed. For significant effects, Sidak’s post hoc adjustment was used. Sample size (*n* = 15), standard deviations, and changes in the average level of the data were assessed for all indicators. The statistical power (SP, as a percentage), based on an alpha level of 0.05, was also performed for all indicators. The significant effect of SP was >80%. The partial eta squared (ηp^2^) was estimated.

To determine any association between oxygen uptake parameters and CF and weight status, Pearson correlation analysis was used. The closer the Pearson correlation coefficient, r, is to either +1 or –1 indicates whether the relationship is positive or negative, respectively. The correlation was considered as strong within the range of 0.5 to 1. Results were considered statistically significant when the *p*-value was <0.05. All values are expressed as mean ± standard deviation.

## 3. Results

### 3.1. Participants’ Characteristics

The subjects’ characteristics are detailed in Table 2. Participants in the two groups had similar height and age, with no significant differences (*p* > 0.05; Table 2). However, body weight in the NW group was lower than in the OW group (ηp^2^ = 0.79, SP > 99%, *p* < 0.05) and the BMI of the NW group was lower than the OW group (ηp^2^ = 0.87, SP > 100%, *p* < 0.05; Table 2).

**Table 2:** Subjects’ characteristics.

Variable	NW Group (*n* = 10)		OW Group (*n* = 10)	
	Mean ± SD	Value Ranges (MIN/MAX)	Mean ± SD	Value Ranges (MIN/MAX)
Age (yr)	10.3 ± 0.94	6/12	9.75 ± 1.03	6/12
Height (m)	149.7 ± 4.8	123/152	147.7 ± 8.1	125/154
Weight (kg)	41.22 ± 5.55 *	25/46	57.87 ± 6.35	40/58
Body mass index (kg/m^2^)	18.32 ± 1.70 *	17/20	26.44 ± 0.57	24/26

NW—normal weight, untrained group; OW—overweight group. Values are means ± standard deviation. * *p* < 0.05 when comparing NW group and OW group.

### 3.2. Oxygen Uptake Parameters

All oxygen uptake parameters are presented in Table 3. The time constant of VO_2_ kinetics during the PTE was significantly shorter in NW than in OW children (ηp^2^ = 0.86, SP > 100%, *p* < 0.05). VO_2_ peak was significantly higher in NW than OW children (ηp^2^ = 0.98, SP > 100%, *p* < 0.05). VO_2_ max was significantly lower in NW than OW children (ηp^2^ = 0.65, SP > 97%, *p* < 0.05).

The NW group had a lower VT max and VE max (ηp^2^ = 0.79, SP > 99%, *p* < 0.05; and ηp^2^ = 0.79, SP > 99%, *p* < 0.05, respectively) than the OW group (Table 3). 

Other oxygen uptake parameters (HRmax, BFmax, RER, and Maximal test power) did not differ significantly between the NW and OW groups (*p* > 0.05; Table 3).

### 3.3. Cognitive Function Evaluation

Cognitive test results were significantly different between the NW and OW groups (Table 4) in the Go/No-Go test (ηp^2^ = 0.72, SP > 100% *p* < 0.05), CSL test (ηp^2^ = 0.61, SP > 99%, *p* < 0.05), and PRO test (ηp^2^ = 0.72, SP > 100%, *p* < 0.05). There was no significant difference between the groups in the 2CRT test (*p* > 0.05).

There were significant, positive, and strong correlations between the Simple Reaction test and VO_2_ peak (r = 0.704; *p* < 0.05), between the Simple Reaction test and VO_2_ max (r = 0.669; *p* < 0.05), and between the Simple Reaction test and the time constant (r = 0.766; *p* < 0.05).

Table 5 shows correlation between oxygen uptake kinetics, VO_2_ peak, and cognitive function tests in NW and OW groups.

## 4. Discussion

Our results indicate that OW children demonstrate lower aerobic capacity and CF parameters than healthy-weight children.

The main measure for assessing aerobic power is the VO_2_ peak, which also reflects endurance capacity. We found a significant difference in VO_2_ peak between NW and OW children. The OW children demonstrated a lower VO_2_ peak than NW children during the PTE test. We presume that OW children got tired, lost motivation, and stopped the test because of leg pain before they reached maximal oxygen uptake. Similar research findings were described by Melendez-Ortega and coworkers [31], who stated that it is challenging to assess VO_2_ parameters in OW children because of their faster rate of tiredness, increased pain sensation, and other effects. The capacity for exercise depends on a lot of factors, but one of the most important is the delivery of O_2_ to working muscles to satisfy their metabolic requirements [32]. Physically active children demonstrate better endurance than OW children. Furthermore, a faster involvement of energy transportation from aerobic metabolism in working muscle and decreased receptivity to muscular fatigue mean that young children can be considered metabolically similar to highly trained sportspeople [33]. However, according to our findings, the higher levels of adiposity among OW children mean the contribution of energy delivery is lower in working muscles.

Obesity and OW may also have an impact on the respiratory system via different mechanisms, including a direct effect of fat in the chest wall to cause structural changes and decrease the volume of the lungs, especially the expiratory reserve volume [34]. Furthermore, because adipose tissue is the largest endocrine organ in the body, when its temperature increases, progressive metabolic changes occur [35]. Endothelial dysfunction, a lower mitochondrial density, and oxygen transfer volume might also be reasons for a lower VO_2_. Initially, obesity can have a negative but not critical effect on full lung volume and residual volume, whereas subsequently, the increased body mass decreases exhalation reserve volume and functional residual volume [34]. These findings demonstrate ongoing risks for OW children and adolescents.

In addition to having a large effect on physical health, OW and obesity may also have an impact on children’s psychosocial health. These children can suffer from depression and low self-respect, and these psychosocial outcomes also affect academic performance [19,20,36].

Neuroimaging studies investigating the relationship between OW and obesity and gray matter report that reward-related regions of the brain in OW/obese children and youths and their healthy-weight peers are different [37].

Groot et al. [38] claim that obese and nonobese children display different brain structures and CFs, and that this may have an impact on appetitive traits. A larger volume of the pallidum has been detected in obese children than in healthy-weight peers. These results support our findings and confirm that cognitive dysfunction may be associated with the pallidum, which plays an important role in cognition. Furthermore, because the prefrontal cortex is important for executive function, regulates limbic reward regions, and is involved in the inhibition of impulsive behaviors [39], this may explain why obese or OW children demonstrate poorer CF than their non-OW peers.

Our findings demonstrate that OW children demonstrate poorer CF compared with NW children. It took longer for the OW children to complete the cognitive tests, and they made more mistakes in the tests than NW children. CF tests measure choice reaction time, working memory, memory, and motor speed. All these functions, especially working memory and memory, influence learning results. The results of this study supplement our previous research and indicate that OW children demonstrate poorer CFs and lower aerobic capacity in all age groups, including young children (6–12 years) and youths (16–19 years) [27]. Our findings are also in line with a report by Datar and colleagues [20] that OW children demonstrate poorer academic achievements. It is known that OW or obese adults display poorer global CFs than NW individuals, and our findings demonstrate that CF in young OW children is worse than in NW children. Early obesity or OW also affects future learning results [28].

This study had some limitations. Our study is limited by the relatively small sample size (*n* = 30), which is common in studies where participants are asked to come to the laboratory several times and performing in the study requires maximum effort. It cost a big withdrawal from the study, as in our case. We studies associations between FC and weight status. It would be beneficial to analyze the impact of such factors as education level, socioeconomical status of parents, nutrition, sleep duration and others for CF, as well. Findings are controversial in the field of CF and AF and overweight and obesity; future studies, especially longitudinal, are needed.

Nonetheless, the present study also has some strengths. Short memory, long memory, and academic achievements are mostly studied assessing CF. We used ANAM4 test battery to assess CF parts, as choice reaction time, scanning, visual tracking, and attention, response inhibition, and visual-motor response timing. The results of our study emphasize that being overweight and obese affect CF and AF at a young age. In addition, it was the first study in Lithuania on this topic with young children.

## 5. Conclusions

In conclusion, OW boys displayed a slower time constant of oxygen uptake, lower VO_2_ peak, and poorer CF compared to normal weight boys. Weight status had an impact on the CF and AF in boys aged 6–12 years. Further studies with bigger sample sizes are required.

## Figures and Tables

**Table 1:** Formulas used for determination of VO_2_ parameters and CF.

Parameter	Formula
Determination of VO_2_ kinetics [30]	$VO_{2}\left(t\right)=VO_{2}\left(b\right)+A_{1}\left(1-e^{-t/\tau_{1}}\right)+A_{2}\left(1-e^{-t/\tau_{2}}\right)$
Determination of VO_2_ peak	The highest VO_2_ within a 20-s period during the PTE.
Determination of CF [29]	#((NumCorr/(NumCorr + Numinc + NumLapse)))

*VO*_2_(*t*)—VO_2_ at any time point; *VO*_2_(*b*)—VO_2_ during 30 s before exercise; *A*_1_—amplitude of the phase I; *A*_2_—amplitude of the phase II of the VO_2_ response; (1 − e^−t/τ^)- $\left(1-e^{-t/\tau_{1}}\right)$ exponential function describing the rate at which the VO_2_ is rising toward the amplitude; *t*—time; τ_1_ and τ_2_—the time constant of phase I and phase II, respectively; #NumCorr—number correct response; Numinc—number of incorrect response; NumLapse—number of no response.

**Table 3:** Parameters of walking exercise among 6–12 years old NW and OW children.

	NW Group	OW Group	*p*
τ1, s	21.73 ± 1.57	33.46 ± 2.9 s	0.00 *
VO_2peak,_ mL/kg/min	49.23 ± 5.75	37.82 ± 3.47	0.00 *
VO_2max,_ L/min	1.61 ± 0.41	2.18 ± 0.18	0.00 *
HR_max_, b/min	195.66 ± 11.93	192.83 ± 9.02	0.66
VE_max_, L/min	56.2 ± 8.14	69.22 ± 1.57	0.00 *
VT_max_, L	1.30 ± 0.2	1.63 ± 0.23	0.05 *
BF_max_, 1/min	44.44 ± 5.48	43.55 ± 5.97	0.29
RER	14.64 ± 1.47	12.57 ± 2.65	0.55
Max test power, W	1.08 ± 0.08	1.03 ± 0.09	0.73

τ1—time constant of VO_2_ kinetics, HR—heart rate, VE—ventilation in minute, BF—frequency of breathing, VT—volume of ventilation, RER—respiratory exchange ratio; NW—normal weight group; OW—overweight group. Values are means ± standard deviation. * *p* < 0.05 when comparing NW group and OW group.

**Table 4:** Cognitive tests results between NW and OW 6–12 years old children.

	NW Group	OW Group	*p*
Go/No-Go,%	93.67 ± 2	90.27 ± 1.83	0.00 *
CSL,%	93.75 ± 3.16	88.95 ± 4.45	0.01 *
2CRT,%	94.4 ± 1.54	93.65% ± 1.5	0.21
PRO,%	93.6 ± 1.7	89 ± 3.7	0.00 *

CSL—Code Substitution–Learning test; 2CRT—2 Choice Reaction test; PRO—Simple Reaction test. NW—normal weight group; OW—overweight group. Values are means ± standard deviation. * *p* < 0.05 when comparing NW group and OW group.

**Table 5:** Correlation between oxygen uptake kinetics, VO_2_ peak, and cognitive function test results.

*Tests*	P.corr, t1, *s*	*p*	P.corr, *VO_2peak_, ml_kg_min*	*p*	NW				OW			
					P.corr, t1, *s*	*p*	P.corr, *VO_2peak_, ml_kg_min*	*p*	P.corr, t1, *s*	*p*	P.corr, *VO_2peak_, ml_kg_min*	*p*
CSL	−0.403	0.05 *	0.592	0.00 *	−0.752	0.00	0.602	0.03 *	−0.109	0.74	−0.403	0.22
Go/No/Go	−0.561	0.00 *	0.415	0.04 *	0.264	0.40	−0.106	0.74	0.201	0.55	−0.086	0.80
2CRT	−0.686	0.00 *	0.504	0.01 *	−0.165	0.60	0.644	0.02 *	0.190	0.57	−0.155	0.64
PRO	−0.767	0.00 *	0.444	0.03 *	−0.321	0.30	0.629	0.02 *	−0.314	0.34	−0.459	0.15

t1—oxygen uptake kinetic, CSL—Code Substitution–Learning test, 2CRT—2Choise Reaction test. PRO—simple reaction time test, P.corr—Pearson correlation, and * *p* < 0.05.

## Data Availability

The data presented in this study are available on request from the corresponding author.
